# Understanding the Perpetuation of Cyberbullying Victimization in Adolescents: The Role of Executive Functions

**DOI:** 10.1007/s10802-022-00926-0

**Published:** 2022-04-19

**Authors:** Aida Morea, Esther Calvete

**Affiliations:** Department of Psychology, University of Deusto, Avenida de las Universidades 24, 48007 Bilbao, Spain

**Keywords:** Cyberbullying victimization, Cyberbullying perpetration, Perpetuation, Executive functions, Depressive symptoms, Adolescents

## Abstract

The consequences of cyberbullying victimization can lead to its perpetuation over time, initiating a vicious cycle. Thus, it is important to identify the factors that contribute to the persistence of victimization. This study aimed to examine some longitudinal mechanisms involved in the perpetuation of cyberbullying victimization in adolescents. The secondary aim was to study the protective role of executive functions (cognitive flexibility and selective attention) in this process, filling a gap in the literature. Sex differences were also studied. The sample consisted of 698 adolescents between 12 and 17 years of age (*M* = 14.59, *SD* = 1.36; 40.83% girls) from the Basque Country (Spain). Adolescents participated in a longitudinal study with three waves, in which 5–6 months elapsed between waves. The participants completed measures of executive functions in wave 1, of cyberbullying perpetration and depressive symptoms in waves 1 and 2, and of cyberbullying victimization in all three waves. The findings indicated that cyberbullying victimization was perpetuated through an increase of cyberbullying perpetration and depressive symptoms. Concerning executive functions, cognitive flexibility attenuated the impact of cyberbullying victimization on cyberbullying perpetration and depressive symptoms, and these in turn predicted lower levels of future victimization. Sex differences revealed that selective attention acted as a risk factor for cyberbullying victimization in girls and as a protective factor in boys. The results highlight the importance of considering mediating mechanisms involved in the perpetuation of cyberbullying victimization as well as the beneficial role played by cognitive flexibility in reducing the stability of cyberbullying victimization.

**Supplementary Information:**

The online version contains supplementary material available at 10.1007/s10802-022-00926-0.

## Introduction

Cyberbullying involves aggressions inflicted through virtual spaces (Kowalski et al., 2014), such as sending or posting derogatory messages or images to damage a person or group. Although traditional bullying and cyberbullying share common features, there are differences between them. For instance, cyberbullying can be carried out at any time and in any place, the victim has nowhere to hide, cyberbullies can attack anonymously, and acts of cyberbullying can reach very large audiences (Kowalski et al., 2014). These characteristics contribute to explaining the high prevalence rates of cyberbullying in several countries. Specifically, the prevalence of cyberbullying varied between 3 and 72% for victimization and between 1 and 41% for perpetration in adolescents (for a systematic review, see Selkie et al., 2016). Among children and adolescents, the mean prevalence was 26.65% for cyberbullying victimization and 24.64% for cyberbullying perpetration (for a systematic review, see Zych et al., 2016).

### Perpetuation of Victimization

The psychological consequences of cyberbullying victimization are severe, especially when the victimization is perpetuated over time (Gámez-Guadix et al., 2015; Rose & Tynes, 2015). Thus, for some adolescents, cyberbullying victimization continues over time, which contributes to increasing the negative outcomes for victims’ mental health (Gámez-Guadix et al., 2015). Consequently, it is important to identify the mechanisms through which victimization is perpetuated over time.

One potential mechanism is that cyberbullying victimization leads to aggressive reactions, and aggressive reactions, in turn, increase the likelihood of new experiences of cyberbullying victimization in adolescents (Gámez-Guadix et al., 2015). Adolescents who experience cyberbullying aggressions from other adolescents could perceive the aggression as inappropriate or unjustified, seek revenge, and react aggressively toward their perpetrators (Calvete et al., 2019), which places them at risk of future victimization (Royuela-Colomer et al., 2018). In fact, a reciprocal and longitudinal relationship has been found between cyberbullying victimization and cyberbullying perpetration in adolescents across several different countries (for a meta-analysis of longitudinal studies, see Marciano et al., 2020).

Another potential perpetuation mechanism of victimization is through internalizing symptoms. Victimization by cyberbullying can lead to depressive moods in the victims (Kowalski et al., 2014), and this in turn makes them more vulnerable to further victimization (Gámez-Guadix et al., 2013). An explanation for this is that adolescents with depressive symptoms may show loneliness and lower social skills, which make them less attractive to peers, create feelings of rejection, and lead to an image of greater vulnerability (Gámez-Guadix et al., 2013). Although research is scarce, in non-clinical samples of adolescents from different countries, depressive symptoms resulting from being a victim predicted new episodes of traditional bullying victimization (Averdijk et al., 2016) and cyberbullying victimization (Gámez-Guadix et al., 2013; Rose & Tynes, 2015).

Therefore, through the above-mentioned mechanisms, adolescents can be caught up in a spiral in which victimization, perpetration, and depressive symptoms affect each other, making it difficult to break the cycle of cyberbullying (Gámez-Guadix et al., 2015). Although research on this issue is scarce, there is an incipient interest in studying the possible mediating effect of depressive symptoms on subsequent peer cyberbullying victimization (Zhang et al., 2020). However, the mediating role of aggressive behavior has not yet been examined, although a recent systematic review of longitudinal studies highlighted the need to fill this gap in the literature (Camerini et al., 2020).

### The Role of Executive Functions in the Reactions to Cyberbullying Victimization

If negative reactions to cyberbullying victimization contribute to its perpetuation, it is important to identify the factors that influence these reactions. One possible factor that may moderate the responses to victimization is executive functions, which are still maturing during adolescence (Poon, 2018). Executive functions are a family of top-down and interrelated psychological processes, which are essential for performing an activity successfully and resolving novel situations when acting automatically or relying on instinct would be imprudent, insufficient, or impossible (Diamond & Ling, 2019). One of the most studied executive functions is cognitive flexibility, which entails the ability to change one’s perspective and/or thoughts about something to adapt to new situations, demands, or problems (Diamond & Ling, 2019). To adopt a new perspective, it is necessary to inhibit the previous one and stimulate another one (Diamond, 2013). Here, selective attention, another executive function, plays an important role, as it involves remaining focused on a particular task or stimulus while avoiding distracting stimuli (Portellano & García, 2014). Selective attention only involves inhibition at the level of attention and does not imply inhibition at other levels, such as behavior, thoughts, and memories (Diamond, 2013). Therefore, selective attention acts as a foundation for cognitive flexibility, allowing the latter to work on new perspectives and thoughts.

Executive functions can be classified in different ways, for example, as cold or hot processes. Cold executive functions are psychological processes that require critical analysis and pure logic without any emotional involvement, while hot executive functions operate in contexts with emotional and motivational stimuli (Poon, 2018). Additionally, there are verbal and visual/non-verbal tasks involved in the evaluation of executive functions, depending on the ability to be assessed (e.g., Portellano & García, 2014).

The executive functions mentioned above could be important resources for adolescents who are victims of cyberbullying. Adequate cognitive flexibility and selective attention could reduce the likelihood of responding to victimization through aggressive responses and depressed mood, thus reducing the perpetuation of the victimization. Nevertheless, despite the close relation between executive functions and social interactions, including online ones (Ybarra & Winkielman, 2012), little is known about the specific role of cognitive flexibility and selective attention in cyberbullying scenarios. Adequate selective attention makes it possible to ignore distracting stimuli (Portellano & García, 2014), whereas adequate cognitive flexibility provides greater ease in issuing alternative responses (Diamond & Ling, 2019). Thus, in cyberbullying scenarios, these executive functions could help the victim by facilitating the generation of adaptive responses to the situation, such as seeking help instead of remaining silent or reacting aggressively, which could then reduce the perpetuation of the victimization. In fact, when children displayed deficits in executive functions in first grade, they were more likely to experience consistent and prolonged bullying victimization over time in sixth grade (Wang & Zhou, 2019). Moreover, deficits in cognitive flexibility were related to bullying victimization and perpetration in preadolescents (Jenkins et al., 2018), and greater self-reported attention problems longitudinally led to more bullying perpetration and victimization in children and adolescents (Ji et al., 2018; Murray et al., 2020).

Furthermore, deficits in both cognitive flexibility and selective attention have been found to be related to depressive symptoms. For instance, in North American adolescents, deficits in cognitive flexibility predicted the first onset of major depressive episodes (Stange et al., 2016), and those with depressive symptoms showed impairments in selective attention (Sommerfeldt et al., 2016). This could be because adolescents can realize that they sometimes have difficulties addressing new situations, such as making new friends (deficits in cognitive flexibility), or find it difficult to concentrate in class (deficits in selective attention). Being aware of these deficits can trigger feelings of hopelessness, sadness, and failure (Hankin et al., 2016), increasing depressive symptoms, particularly among girls (Keyes et al., 2019). Therefore, adequate executive functions could help reduce the likelihood that victims of cyberbullying will react with a depressive mood. As indirect evidence for this hypothesis, other variables closely associated with executive functions, such as mindfulness (Diamond & Ling, 2019) and resilience (Taylor & Ruiz, 2019), buffer the negative impact of cyberbullying victimization on the development of depressive symptoms in adolescents (Faura-Garcia et al., 2021; Santos et al., 2021). Further, attention deficit hyperactivity disorder (ADHD) symptoms, which are associated with deficits in executive functions (Kofler et al., 2019), led to depressive symptoms in clinical and non-clinical children and adolescents from several countries, with traditional bullying having a moderating role in some studies and a mediating one in others (for a systematic review, see Simmons & Antshel, 2021).

### Sex Differences in Longitudinal Relationships Between Cyberbullying, Depressive Symptoms, and Executive Functions

Studies on possible sex differences in the above-mentioned mechanisms are scarce. In one study, the effect of cyberbullying perpetration on cyberbullying victimization was cross-sectionally stronger for boys than girls (Kowalski et al., 2012). However, a longitudinal study indicated that cyberbullying victimization predicted cyberbullying perpetration in girls but not in boys, whereas cyberbullying perpetration did not predict cyberbullying victimization in girls or boys (Festl & Quandt, 2016).

Regarding depressive symptoms, mixed evidence has also been reported in non-clinical adolescents. Depressive symptoms longitudinally predicted cyberbullying victimization, but only in boys (Chu et al., 2019). For traditional bullying, depressive symptoms at age 15 predicted victimization at age 17 only in girls, but depressive symptoms at age 15 did not predict perpetration at age 17 in either sex (Kaltiala-Heino et al., 2010).

Concerning executive functions, there were no sex differences in the predictive associations between self-reported attention problems and traditional bullying perpetration and victimization in preadolescents and adolescents (Murray et al., 2020). Cognitive flexibility was cross-sectionally and negatively related to traditional bullying victimization only in boys and with traditional bullying perpetration only in girls in preadolescents (Jenkins et al., 2018).

### Current Study

In this study, a mediated moderation model was tested. Thus, the first objective was to analyze the potential mediating role of cyberbullying perpetration and depressive symptoms in the perpetuation of cyberbullying victimization in adolescents. Although no studies were found that indicate the existence of such mediational mechanisms, bidirectional and longitudinal relationships have been found between cyberbullying victimization and depressive symptoms (Gámez-Guadix et al., 2013) and between cyberbullying victimization and cyberbullying perpetration (Marciano et al., 2020). Thus, it was hypothesized that cyberbullying perpetration and depressive symptoms would play a mediating role in the perpetuation of cyberbullying victimization.

A second objective was to examine the role of executive functions in reactions to cyberbullying victimization and therefore in the perpetuation of victimization. Specifically, we hypothesized that two executive functions (cognitive flexibility and selective attention) would attenuate the negative impact of cyberbullying victimization on the prediction of cyberbullying perpetration, depressive symptoms, and the perpetuation of cyberbullying victimization. In the present study, two cold and visual/non-verbal tasks were selected for the evaluation of cognitive flexibility and selective attention, with the aim of not involving any emotional or verbal component in the task that could influence the relationships to be studied.

Finally, the third objective was to study sex differences in the predictive model. Previous studies highlighted sex differences in several of the longitudinal relationships that were examined in this study, but there is not a clear consensus on the direction of these differences. Therefore, we did not hypothesize any specific finding for sex differences.

## Methods

### Participants

The sample included 698 adolescents between 12 and 17 years of age (*M* = 14.59, *SD* = 1.36, 285 girls and 413 boys). All participants completed at least one of the three waves of the study, which were each separated by 5–6 months. Of the total sample, 12.2% did not participate in Wave 1 (W1), 10.3% in Wave 2 (W2), and 15.3% in Wave 3 (W3). The attrition rates were due to not attending class on the days of measurement. The adolescents were from two public and three private high schools in the Basque Country (Spain). Socioeconomic status was determined based on the criteria of the level of work occupation by the Spanish Society of Epidemiology and Family and Community Medicine (2000). The distribution of socioeconomic status was as follows: 12.5% low (unskilled workers), 12.3% low-medium (skilled and semi-skilled workers), 26.4% medium (administrative employees, administrative and financial management), 21.5% high-medium (managers of companies with less than 10 employees, professions associated with a first-cycle university degree, technicians), and 18.8% high (managers of companies with 10 or more employees, professions associated with second- and third-cycle university degrees). For 8.6% of the participants, there was no information on socioeconomic status.

The power analysis carried out with the a priori sample size calculator (Soper, 2021) showed that when the anticipated effect size is 0.1 and the desired statistical power is 80%, the minimum sample size for the model structure is ≈ 700.

### Measures

The Changes, Cognitive Flexibility Test (Seisdedos, 2004) was used to assess cognitive flexibility. It is a performance test composed of 27 trials. In each trial, the participant aims to detect the changes that can occur between three polygons, which can change in terms of their number of sides, size, and color. There are two circles with specific symbols (arrows, lines, triangles, and/or squares) between the polygons, which determine how the right polygon changes from the left polygon. The meaning of these symbols appears in a table. To answer the test, the participants should choose an option (A, B, C, or D) indicating whether or not the changes in the circles (i.e., the symbols) have been fulfilled. The participants had 7 min to complete the task, during which they had to answer as many trials as possible without making mistakes. The Changes has been shown to have good criterion and construct validity as well as appropriate internal consistency (Seisdedos, 2004). In this study, the Cronbach’s α coefficient was 0.95.

A Spanish adaptation (Seisdedos, 2012) of the d2 Test (Brickenkamp, 1962) was employed to assess selective attention. The d2 Test has 14 rows, each one made up of “d” and “p” letters and with different numbers of lines (from one to four) around each letter. For each row, the participants had to process as many as letters as possible, crossing out every instance of the letter “d” with two lines anywhere without making mistakes for 20 s. The d2 Test has good construct validity and adequate internal consistency (Seisdedos, 2012). The test provides several indicators. In this study, the total score (TS) of the test was used. The TS is a combination of three indicators: the total number of items processed (IP); the error of commission, which represents the number of irrelevant items crossed out (CE); and the error of omission, which represents the number of relevant items present but not crossed out (OE). The TS is determined as follows: TS = IP – (CE + OE). In the present study, the Cronbach’s α coefficients were 0.94 for IP, 0.97 for CE, and 0.95 for OE.

The Cyberbullying Questionnaire (CBQ; Calvete et al., 2010; Gámez-Guadix et al., 2014) was employed to assess cyberbullying (victimization and perpetration). The CBQ has two different scales, each composed of nine items. One scale evaluates cyberbullying victimization (e.g., “Post or send pictures of me that can be humiliating”), and the other evaluates cyberbullying perpetration (e.g., “Send threatening or insulting messages to other people”) in the last 6 months. The CBQ is rated on a four-point scale, ranging from 0 (*never*) to 3 (*5 or more times*). Higher scores indicate a higher level of cyberbullying. The Spanish version of the CBQ has displayed good internal consistency and adequate factorial and convergent validity (Gámez-Guadix et al., 2014). In this study, both scales of the CBQ showed non-normal distribution. Thus, MPLUS 8.2 software and the WLSMV (weighted least squares means and variance adjusted) estimation method were used to calculate the factor loadings and estimate ordinal α in both scales of the questionnaire. To calculate ordinal α, the Excel document created by Domínguez (2012) was employed. The ordinal’s α coefficients in this study were 0.90 at both W1 and W2 and 0.91 at W3 for the cyberbullying victimization scale, while these coefficients were 0.93 at W1 and 0.88 at W2 for the cyberbullying perpetration scale.

The Center for Epidemiological Studies Depression Scale (CES-D; Radloff, 1977) was used to evaluate depressive symptoms. This scale has 20 items and is rated on a four-point scale, ranging from 0 (*practically never*) to 3 (*most of the time*). Some of the items are “I felt annoyed by thing that usually do not bother me” and “I enjoyed life.” The higher the score, the higher the depressive symptoms. The factor structure of the Spanish version of the CES-D has been confirmed, and it has demonstrated excellent psychometric properties (Calvete & Cardeñoso, 1999). The Cronbach’s α coefficients in this study were 0.87 at W1 and 0.88 at W2.

### Procedure

This study is part of a broader project focused on the role of executive functions in psychological problems in adolescents. Various high schools in the Basque Country (Spain) were contacted, and a cover letter about the present study was sent to them. Five high schools agreed to participate. The informed consent form was sent to parents of adolescents, who had the option of refusing to allow their children to take part in the study (non-participation rate = 1.13%). Additionally, the adolescents were informed that their participation was voluntary and that their answers were confidential and anonymous. All of them participated in the study. They completed the tests in their classrooms during the three waves. At W1, adolescents took around 45 min to finish the Changes, d2 Test, CBQ, and CES-D. At W2, they completed CBQ and CES-D in 15−20 min, and at W3 the adolescents only completed the victimization scale of CBQ, taking about 10 min to finish it. The Changes and d2 tests were administered first. Aiming to maintain the motivation and attention of the adolescents, after the administration of the Changes and d2 tests, they were informed in all waves that they would take part in a raffle for gift vouchers. The Ethics Committee of the University of Deusto approved this study (ETK-5/18–19).

Participants who completed W2 and/or W3 and those who only completed W1 were compared with regard to all W1 variables. Those who only completed W1 scored significantly higher on W1 cyberbullying perpetration (*M* = 3.88, *SD* = 4.94 vs. *M* = 0.95, *SD* = 2.03; *t*(605) = 2.37, *p* = 0.032, *d* = 0.78) and W1 depressive symptoms (*M* = 21.74, *SD* = 10.28 vs. *M* = 15.78, *SD* = 9.79; *t*(567) = 2.40, *p* = 0.017, *d* = 0.59). There were no differences in the rest of the W1 variables.

### Data-analysis Plan

LISREL 8.8 software was used to test the hypotheses of the study. The Little’s test of Missing Completely at Random (MCAR) was statistically significant, χ^2^ (280, *N* = 698) = 565.34, *p* < 0.001, indicating that missingness was not random. Therefore, the full information maximum likelihood (FIML) approach was employed, and auxiliary variables were included in the analyses to reduce the nonresponse bias. In this study, two variables were used as auxiliary variables because they were correlates of missingness: age and ruminative style. Although the ruminative style measure was not part of this study, this measure was available to us because the study was part of a larger project. Ruminative style was measured by the Ruminative Responses subscale from the Children’s Response Styles Scale (CRSS; Ziegert & Kistner, 2002). This subscale is a self-report questionnaire of children’s tendency to ruminate in response to sad moods and presented a Cronbach’s α coefficient of 0.71 in this study. Following the procedures indicated by Enders (2010), both auxiliary variables (age and ruminative style) were included in the model and modeled to covary between them, with the rest of the predictor variables, and with the residual term. The inclusion of auxiliary variables in the model improves the chances of random missingness (Enders, 2010).

To evaluate the goodness of the model fit, the comparative fit index (CFI), Tucker–Lewis index (TLI), and root-mean-square error of approximation (RMSEA) were employed. For longitudinal research, CFI and TLI values of 0.95 or higher show very good fit, and RMSEA values lower than 0.08 indicate adequate fit (Little, 2013). A mediational model was tested. This involved paths from W1 cyberbullying victimization to W2 depressive symptoms and cyberbullying perpetration and from W2 depressive symptoms and cyberbullying perpetration to W3 cyberbullying victimization. In addition, the model included the moderating role of executive functions (i.e., cognitive flexibility and selective attention) in the relationships between W1 cyberbullying victimization and W2 cyberbullying victimization, cyberbullying perpetration, and depressive symptoms. To plot the moderation trajectories, the two-way and three-way linear interactions plotters available on Jeremy Dawson’s “Interpreting interaction effects” website were employed (Dawson, 2021).

Bootstrapping was carried out to test the significance of the mediation paths. First, 5000 bootstrapping samples were generated from the original dataset by random sampling with replacement. In each sample, the covariance matrix was calculated. Second, the path analysis model was performed 5000 times with these 5000 bootstrap covariances to produce 5000 estimations of each path coefficient. Third, LISREL’s saved output of the estimations of each path coefficient was employed to calculate the indirect effect. Finally, the 95% CI for the estimated indirect effect was analyzed, with the indirect effect considered significant at the 0.05 level if the 95% confidence level did not contain zero (Shrout & Bolger, 2002).

## Results

### Descriptive Statistics and Correlation Between Variables

The descriptive statistics and correlation between the variables of the study are shown in Table 1. All associations between variables were significant, except for executive functions (i.e., cognitive flexibility and selective attention), which only correlated between themselves. Notably, there were high coefficients between cyberbullying victimization and cyberbullying perpetration in the same waves. All significant relationships were positive.

**Table 1:** Descriptive Statistics and Correlation Between Variables

	1	2	3	4	5	6	7	8	9	*M*	*SD*
1. W1 CB victimization	1									1.36	2.27
2. W1 CB perpetration	0.68**	1								1.02	2.20
3. W1 Depressive symptoms	0.39**	0.22**	1							15.95	9.84
4. W1 Selective attention	−0.01	0.05	−0.06	1						411.03	83.40
5. W1 Cognitive flexibility	0.01	0.01	−0.03	0.47**	1					9.82	5.60
6. W2 CB victimization	0.47**	0.24**	0.33**	0.03	0.02	1				1.29	2.17
7. W2 CB perpetration	0.32**	0.36**	0.18**	0.07	−0.04	0.60**	1			0.91	1.68
8. W2 Depressive symptoms	0.31**	0.15**	0.68**	−0.03	0.01	0.38**	0.22**	1		15.72	9.53
9. W3 CB victimization	0.31**	0.15**	0.22**	0.07	0.02	0.44**	0.31*	0.30**	1	1.16	2.07

*W1* wave 1, *W2* wave 2, *W3* wave 3, *CB* cyberbullying

^*^p < 0.05. **p < 0.01

### Predictive Model

The predictive model was comprised of cross-sectional associations between variables (cyberbullying victimization, cyberbullying perpetration, depressive symptoms, selective attention, and cognitive flexibility), autoregressive paths for cyberbullying victimization from W1 to W2 and from W2 to W3, and autoregressive paths for cyberbullying perpetration and depressive symptoms from W1 to W2. Moreover, the model included paths from two interaction terms (cyberbullying victimization x selective attention at W1 and cyberbullying victimization x cognitive flexibility at W1) to W2 variables. Finally, sex was added to the model to control its potential relationship with the other variables. This model displayed excellent fit indices (FIML χ^2^ (20, *N* = 698) = 54.25, *p* < 0.001, RMSEA = 0.05 (90% CI [0.03, 0.07]), TLI = 0.99, and CFI = 0.99).

**Fig. 1:**
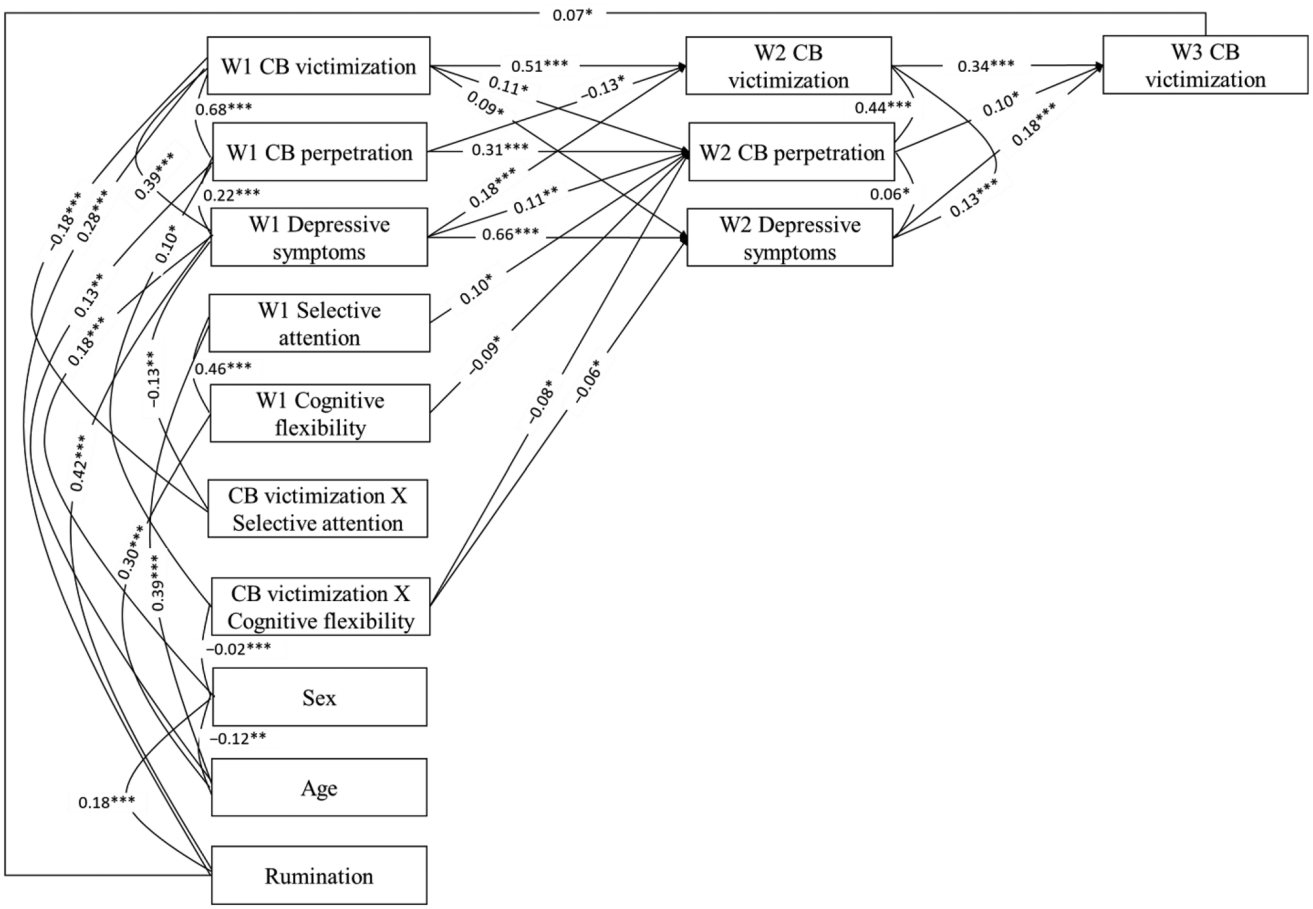
Predictive Model for Cyberbullying Victimization. Note. Standardized values are displayed. *W1* wave 1, *W2* wave 2, *W3* wave 3, *CB* cyberbullying. *p < 0.05. **p < 0.01. ***p < 0.001

Figure 1 shows the standardized parameters of the predictive model. All the autoregressive paths were statistically significant (*p* < 0.001) and had small-to-large coefficients, indicating the stability of these variables over time. The predictive associations from W1 cyberbullying victimization to W2 depressive symptoms and from W1 depressive symptoms and W2 cyberbullying victimization were statistically significant and positive, indicating reciprocity between victimization and depressive symptoms. In addition, W2 depressive symptoms predicted higher W3 cyberbullying victimization. These effects were small. W1 cyberbullying victimization predicted a small increase of W2 cyberbullying perpetration. Nevertheless, cyberbullying perpetration displayed mixed effects, as W1 cyberbullying perpetration predicted a decrease of W2 cyberbullying victimization, whereas W2 cyberbullying perpetration predicted an increase of W3 cyberbullying victimization, both with small coefficients. W1 depressive symptoms predicted higher W2 cyberbullying perpetration with a low coefficient. Both executive functions predicted W2 cyberbullying perpetration, but selective attention predicted a higher score and cognitive flexibility a lower score, both with small coefficients.

**Fig. 2:**
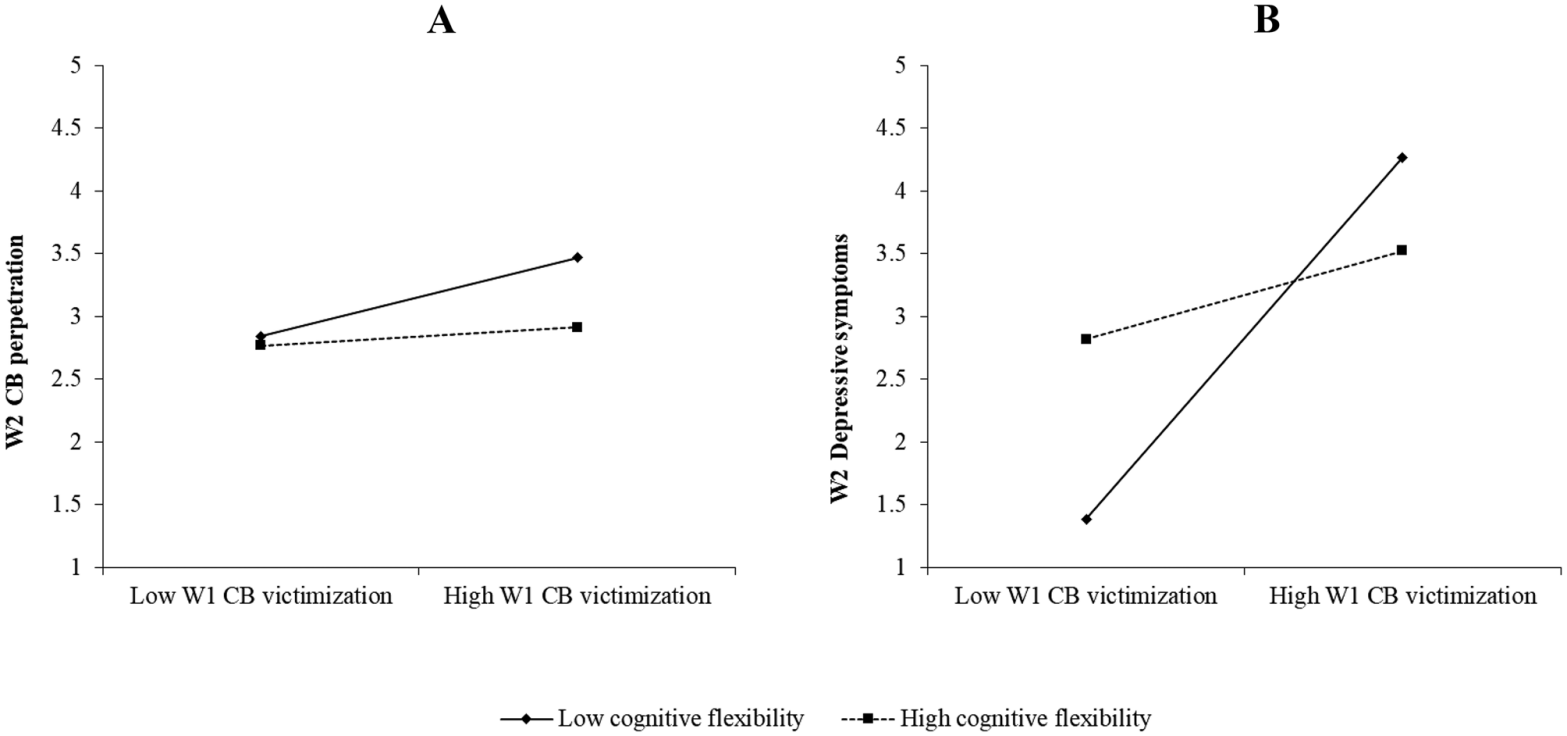
Two-Way Interactions Between W1 Cyberbullying Victimization and Cognitive Flexibility for W2 Cyberbullying Perpetration and W2 Depressive Symptoms. Note. *W1* wave 1, *W2* wave 2, *CB* cyberbullying

Selective attention did not moderate the effects of cyberbullying in any W2 variable. However, cognitive flexibility moderated the associations between cyberbullying victimization and W2 cyberbullying perpetration and depressive symptoms, with small effects. Figure 2 displays the form of these interactions for adolescents who scored low (one *SD* below the mean) and high (one *SD* above the mean) in W1 cyberbullying victimization and cognitive flexibility. Figure 2A shows that the association between cyberbullying victimization and cyberbullying perpetration was higher when cognitive flexibility was low. The slope of the association was positive and statistically significant when cognitive flexibility was low (*B* = 0.31, *t*(694) = 2.75, *p* = 0.006) and non-significant when it was high (*B* = 0.07, *t*(694) = 0.64, *p* = 0.522). Similarly, Fig. 2B shows that the predictive association between cyberbullying victimization and depressive symptoms was higher among adolescents who were low in cognitive flexibility. Here again, the slope of the relationship was positive and statistically significant when cognitive flexibility was low (*B* = 1.44, *t*(694) = 2.68, *p* = 0.007) but non-significant when it was high (*B* = 0.35, *t*(694) = 0.84, *p* = 0.403).

Bootstrapping analysis was employed to analyze the seven mediating paths proposed by the predictive model. The 95% confidence intervals and mean indirect effects for possible mediations in the predictive model are included as Supplementary material. All the indirect pathways were statistically significant, as they did not contain zero. Thus, cyberbullying perpetration at W2 mediated the relationship between several variables at W1 (cyberbullying victimization, depressive symptoms, executive functions, and cyberbullying victimization x cognitive flexibility interaction term) and cyberbullying victimization at W3. Specifically, W1 cyberbullying victimization, W1 depressive symptoms, and W1 selective attention led to higher levels of W2 cyberbullying perpetration, which, in turn, predicted higher W3 cyberbullying victimization. Cognitive flexibility at W1 and cyberbullying victimization x cognitive flexibility interaction term were indirectly associated with W3 cyberbullying victimization through levels of W2 cyberbullying perpetration. Finally, W2 depressive symptoms mediated the relationship between two variables at W1 (cyberbullying victimization and cyberbullying victimization x cognitive flexibility interaction term) and W3 cyberbullying victimization. Cyberbullying victimization at W1 predicted higher levels of depressive symptoms at W2, which, in turn, predicted high cyberbullying victimization at W3. Cyberbullying victimization x cognitive flexibility interaction term predicted W3 cyberbullying victimization through levels of W2 depressive symptoms.

### Sex Differences in the Predictive Model

Sex differences in the study variables are presented as Supplementary material. Girls scored higher than boys on W1 and W2 depressive symptoms. The effect sizes were moderate and significant (*p* < 0.001). There were no sex differences in the other variables.

Next, we examined whether the predictive model was invariant for boys and girls. An unconstrained model with all the parameters estimated in each sample was calculated. This model presented good fit indices (FIML χ^2^ (32, *N* = 698) = 71.39, *p* < 0.001, RMSEA = 0.06 (90% CI [0.04, 0.08]), TLI = 0.98, and CFI = 0.99). The unconstrained model was compared with a constrained model in which longitudinal paths were constricted to be equal across both subsamples. This imposition increased the χ^2^ value significantly (Δχ^2^ [24, *N* = 698] = 90.32; *p* < 0.001), showing that the model was different for boys and girls. Thus, individual paths were examined to find where the differences were.

**Fig. 3:**
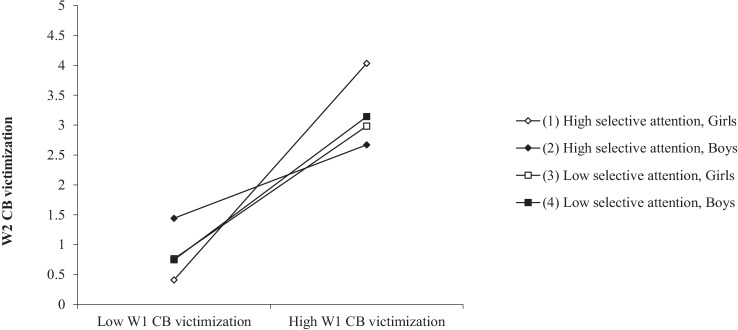
Three-Way Interaction Between W1 Cyberbullying Victimization and Selective Attention for W2 Cyberbullying Victimization in Girls and Boys. Note. *W1* wave 1, *W2* wave 2, *CB* cyberbullying

The path from W1 cyberbullying victimization x selective attention interaction term to W2 cyberbullying victimization was significantly different for girls and boys (*B* = 0.43 [*SE* = 0.20]; *p* = 0.032, vs *B* = −0.29 [*SE* = 0.11], *p* = 0.007; Δχ^2^ [1, *N* = 698] = 8.92; *p* = 0.003). These tendencies are plotted in Fig. 3. In girls, there was higher stability of victimization in those who scored higher in selective attention, whereas in boys the opposite was true. When selective attention was low, both sexes displayed similar stability. The slope of the association was positive and statistically significant when selective attention was high in girls (*B* = 1.81, *t*(690) = 8.36, *p* < 0.001) and in boys (*B* = 0.61, *t*(690) = 3.55, *p* < 0.001), and also when it was low in girls (*B* = 1.11, *t*(690) = 5.93, *p* < 0.001) and in boys (*B* = 1.20, *t*(690) = 8.93, *p* < 0.001).

The path from W2 cyberbullying perpetration to W3 cyberbullying victimization was significantly stronger in girls than in boys (*B* = 0.34 [*SE* = 0.07]; *p* < 0.001, vs *B* = 0.03 [*SE* = 0.09], *p* = 0.754; Δχ^2^ [1, *N* = 698] = 6.05; *p* = 0.014). Finally, the path from W2 depressive symptoms to W3 cyberbullying victimization was significantly stronger in girls than in boys (*B* = 0.07 [*SE* = 0.01]; *p* < 0.001, vs *B* = 0.01 [*SE* = 0.02], *p* = 0.550; Δχ^2^ [1, *N* = 698] = 7.94; *p* = 0.005). The other paths were similar for boys and girls.

## Discussion

Previous research indicates that for many adolescents cyberbullying victimization can be perpetuated over time (Rose & Tynes, 2015), thereby increasing the risk of negative outcomes, such as psychological problems (Gámez-Guadix et al., 2015). The current study aimed to identify some of the mechanisms involved in the perpetuation of cyberbullying victimization over time and examined the role of executive functions in this process.

In line with our hypothesis, the findings indicate that cyberbullying victimization was perpetuated over time through an increase in cyberbullying perpetration and depressive symptoms, which is consistent with some theoretical approaches, such as the general aggression model (Anderson & Bushman, 2002). Thus, adolescents who are victims of cyberbullying can develop depressive symptoms (sadness, tiredness, loneliness) and/or react aggressively. These outcomes could make them less attractive to peers and increase the likelihood of future cyberbullying victimization (Gámez-Guadix et al., 2013). However, this result had a limitation in that a negative longitudinal path was also observed between cyberbullying perpetration at W1 and cyberbullying victimization at W2. Therefore, the results regarding the longitudinal relationship between cyberbullying perpetration and cyberbullying victimization were mixed, as they were opposite from W1 to W2 and from W2 to W3. The above-mentioned literature supported the notion that cyberbullying perpetration predicts an increase in cyberbullying victimization (Marciano et al., 2020; Royuela-Colomer et al., 2018). Nevertheless, one study found that traditional bullying perpetration predicted lower bullying victimization systematically in non-clinical Korean adolescents (Choi & Park, 2021). The authors of that study interpreted this finding to indicate that, by bullying others, the bullies would have more resources to escape victimization, which could also occur in cyberbullying. Nonetheless, due to the contrary longitudinal relationships found in this study, the findings should be considered with caution.

Interestingly, and consistent with previous research on preadolescents (Zhang et al., 2020), this study found that depressive symptoms not only predicted more victimization but also the perpetration of cyberbullying. Delving into these relationships, it was found that cyberbullying perpetration mediated the association between depressive symptoms and cyberbullying victimization. It has been proposed that individuals with depressive symptoms are at a higher risk of experiencing interpersonal problems (Kochel et al., 2012), and this could include both cyberbullying perpetration and victimization (Zhang et al., 2020).

Regarding the role of executive functions, the results indicated that only cognitive flexibility moderated the predictive associations from cyberbullying victimization to both cyberbullying perpetration and depressive symptoms. Namely, the association between W1 cyberbullying victimization and W2 cyberbullying perpetration and depressive symptoms was lower when cognitive flexibility was high. Consequently, cognitive flexibility displayed a protective role against the development of cyberbullying behaviors and depressive symptoms after victimization, reducing future cyberbullying victimization. Thus, cognitive flexibility seems to contribute to breaking the cycle of perpetuation of victimization by decreasing cyberbullying perpetration and depressive symptoms as reactions to the victimization. A possible explanation for this could be that adolescents who are high in cognitive flexibility respond to cyberbullying victimization with more helpful strategies (e.g., block the perpetrator on all social media and look for help; Kowalski et al., 2014), which could reduce the negative impact of cyberbullying and, consequently, help them to avoid the perpetuation of victimization over time. Another finding of this study was that cyberbullying perpetration mediated the relationship between cognitive flexibility and cyberbullying victimization. Specifically, good cognitive flexibility predicted lower cyberbullying perpetration, which in turn led to lower victimization. Good cognitive flexibility leads to the consideration of different alternatives to a situation or problem (Diamond & Ling, 2019), which can lead the individual to avoid aggressive behavior and, consequently, reduce future victimization.

Selective attention did not attenuate the negative impact of cyberbullying victimization on the prediction of cyberbullying perpetration, depressive symptoms, and the perpetuation of cyberbullying victimization. Furthermore, selective attention played a dysfunctional role, as better selective attention led to higher cyberbullying perpetration, which, in turn, predicted higher cyberbullying victimization. This finding was unexpected since previous research involving a non-clinical sample of Swiss adolescents concluded the opposite, that is, that attention problems led to greater perpetration (Murray et al., 2020). Accordingly, future research should attempt to replicate these findings and examine the circumstances under which selective attention is maladaptive. It is possible, for example, that selective attention leads to forms of attentional biases to potential threats in ambiguous situations in some adolescents. These attentional biases, in turn, could increase aggression and hostile intent attributions (Miller & Johnston, 2019). Future research should explore this potential mechanism in cyberbullying contexts. Moreover, in the current study, selective attention was assessed as a cold executive function, so future studies should examine whether selective attention to emotional stimuli (i.e., assessed as a hot process) plays a different role than cold selective attention in adolescents who experience victimization.

The last objective was to analyze sex differences in the predictive model. The results indicated that there were sex differences in the moderating role of selective attention in the stability of cyberbullying victimization from W1 to W2. In girls, it seems that selective attention acted as a risk factor increasing the stability of cyberbullying victimization, whereas in boys selective attention played a protective role. A possible explanation for this may be related to the fact that, as in previous research (Ghandour et al., 2019), the girls experienced more depressive symptoms than boys, which could influence the functionality of their selective attention. Girls who are high in selective attention could focus greater attention on the negative aspects of internalizing symptoms, which would make them more vulnerable to future victimization. However, more research is needed in this field.

Meanwhile, the paths from W2 cyberbullying perpetration and depressive symptoms to W3 cyberbullying victimization were stronger in girls than in boys. These mechanisms could contribute to the higher stability of victimization among girls. The stronger association between perpetration and victimization in girls is contrary to previous findings in German adolescents (Festl & Quandt, 2016), whereas the stronger association between depressive symptoms and victimization is consistent with previous studies on Finnish adolescents involving non-clinical samples (Kaltiala-Heino et al., 2010). Because girls suffer more depressive symptoms than boys (Ghandour et al., 2019), they could be more vulnerable to victimization by peers. Nonetheless, these findings should be considered with caution since they only occurred between W2 and W3 and not between W1 and W2.

This study has some limitations that provide opportunities for future research. First, the predictive model included victimization and perpetration of cyberbullying. However, it would have also been interesting to study the role of cyberbystanders in this model, as they can influence cyberbullying situations by helping the victim, reinforcing the aggressor, and/or worsening the situation with their passivity (Machackova, 2020). Thus, future research could replicate this work including the experience of witnessing cyberbullying and analyze its relationship with executive functions and internalizing symptoms. Second, the present study focused on two cold executive functions: cognitive flexibility and selective attention. Future studies could examine whether other cold executive functions, such as working memory and inhibitory control, are longitudinally related to cyberbullying, both of which have been studied in relation to bullying (Medeiros et al., 2016). Furthermore, given the unexpected results regarding selective attention, future studies should examine the role of hot executive functions, which include emotional components. There is a gap in the research on cyberbullying and executive functions, so their relationships require further study. Third, the only internalizing symptoms that were evaluated were depressive symptoms. It would be useful to consider other internalizing symptoms, such as anxiety, as well as other psychological problems, including eating disorders and self-injury behaviors, which have been identified as potential outcomes of cyberbullying victimization in non-clinical Spanish adolescents (Faura-Garcia et al., 2021; Marco & Tormo-Irun, 2018). Fourth, executive functions were only measured at the beginning of the study. However, they could have changed over the course of the study and been influenced by other study variables, such as victimization and depressive symptoms. Stressful situations (e.g., victimization) and some depressive symptoms (e.g., loneliness and sadness) can influence executive functions and the prefrontal cortex, so that they can mimic an executive function disorder, such as ADHD (Diamond, 2013). Finally, the sample was a community sample, and the results need to be replicated in clinical samples of adolescents. In fact, although missingness was low, the adolescents who failed to respond at W2 and W3 scored higher on cyberbullying perpetration and depressive symptoms. The power analysis indicated that the required sample size was ≈ 700 participants, which could not be met because the loss of participants was between 10.3% and 15.3% in each wave. Moreover, the sample only comes from the Basque Country (Spain). Therefore, the findings of the study are not generalizable to the adolescent populations in other geographical areas and should be considered with caution.

Despite these limitations, the current study also has several strengths. First, it used a longitudinal design with three waves, which included mediation and moderation relationships. With this design, the study contributes to determining the mediating role of cyberbullying perpetration and depressive symptoms in the perpetuation of cyberbullying victimization. In addition, the study fills a gap in the literature by examining the moderating role of cognitive flexibility and selective attention when cyberbullying victimization occurs. The findings contribute to knowledge on the longitudinal relationships between executive functions, cyberbullying, and depressive symptoms while providing opportunities for future research to examine other possible mediating and/or moderating mechanisms between these variables (e.g., personality traits and psychological symptoms). Finally, despite the fact that the discrepancy in the results between performance and self-report measures has been discussed in the literature (Teglasi et al., 2017), in this study significant relationships were found between measures obtained with different methods (i.e., performance tasks to assess executive functions and self-report questionnaires for the rest of the variables). This provides support to the findings of this study since the performance tasks are less biased by variables such as depressive symptoms, which could lead an adolescent to minimize his or her skills in the use of executive functions.

To conclude, the current study claims that cyberbullying perpetration and depressive symptoms contribute to perpetuating cyberbullying victimization over time. This emphasizes the need to break the cyberbullying victimization–cyberbullying perpetration and cyberbullying victimization–depressive symptoms cycles to reduce cyberbullying victimization. Interventions targeting cyberbullying perpetration and depressive symptoms are essential for adolescents who are victims of cyberbullying.

Additionally, the study also highlights that cognitive flexibility plays an important role in buffering the negative impact of being victimized by cyberbullying on the development of cyberbullying perpetration and depressive symptoms. Because of this protective role of cognitive flexibility, training in this executive function should be included in cyberbullying prevention and intervention programs. This could contribute to diminishing the persistence of victimization by reducing cyberbullying perpetration and depressive symptoms. In fact, cognitive flexibility training is often included as part of some cognitive-behavioral therapy programs, such as the Unified Protocol-Adolescents, for the treatment of depression in adolescents (Ehrenreich-May et al., 2017). The possible benefits of its extension to traditional bullying and cyberbullying programs should be considered in future research.

## Supplementary Information

Below is the link to the electronic supplementary material.

Supplementary file1 (DOCX 20 KB)

## Data Availability

This manuscript’s data will be deposited at OSF (https://osf.io/HS45K).
