# Supplementary material for: Understanding the Perpetuation of Cyberbullying Victimization in Adolescents: The Role of Executive Functions
Source: Res Child Adolesc Psychopathol. 2022 Apr 19;50(10):1299–311. doi: 10.1007/s10802-022-00926-0 (PMC9606089; doi:10.1007/s10802-022-00926-0)
Supplement: Supplementary file 1 — Supplementary file1 (DOCX 20 KB) [file 10802_2022_926_MOESM1_ESM.docx]

**Supplementary Material**

*Confidence Intervals and Mean Indirect Effects for Possible Mediations in the Predictive Model*

|  | Mean indirect effect | 95% CI | |
| --- | --- | --- | --- |
|  |  | Lower | Upper |
| W1 CB victimization 🡪 W2 CB perpetration 🡪 W3 CB victimization | .01 | .01 | .01 |
| W1 Depressive symptoms 🡪 W2 CB perpetration 🡪 W3 CB victimization | .00 | .00 | .00 |
| W1 Selective attention 🡪 W2 CB perpetration 🡪 W3 CB victimization | .01 | .01 | .01 |
| W1 Cognitive flexibility 🡪 W2 CB perpetration 🡪 W3 CB victimization | −.02 | −.02 | −.01 |
| W1 CB victimization x cognitive flexibility 🡪 W2 CB perpetration 🡪 W3 CB victimization | −.01 | −.01 | −.00 |
| W1 CB victimization 🡪 W2 Depressive symptoms 🡪 W3 CB victimization | .03 | .03 | .03 |
| W1 CB victimization x cognitive flexibility 🡪 W2 Depressive symptoms 🡪 W3 CB victimization | −.02 | −.02 | −.02 |

*Note*. 5000 bootstrap samples. All the indirect pathways were statistically significant. W1 = wave 1; W2 = wave 2; W3 = wave 3; CB = cyberbullying.

*Sex Differences in the Variables of the Study*

|  | Girls (*n* = 285) | | Boys (*n* = 413) | |  |  |  |  |
| --- | --- | --- | --- | --- | --- | --- | --- | --- |
|  | *M* | *SD* | *M* | *SD* | *t* | *df* | *p* | *d* |
| W1 CB victimization | 1.45 | 2.14 | 1.30 | 2.36 | 0.78 | 605 | .437 | 0.07 |
| W1 CB perpetration | 0.98 | 1.92 | 1.05 | 2.37 | −0.38 | 605 | .706 | −0.03 |
| W1 Depressive symptoms | 17.81 | 10.88 | 14.67 | 8.85 | 3.65 | 428 | <.001 | 0.32 |
| W1 Selective attention | 412.54 | 78.54 | 410.02 | 86.58 | 0.36 | 598 | .717 | 0.03 |
| W1 Cognitive flexibility | 9.89 | 5.21 | 9.77 | 5.86 | 0.27 | 569 | .786 | 0.22 |
| W2 CB victimization | 1.40 | 2.32 | 1.21 | 2.07 | 1.06 | 617 | .289 | 0.09 |
| W2 CB perpetration | 0.85 | 1.70 | 0.96 | 1.67 | −0.74 | 616 | .462 | −0.06 |
| W2 Depressive symptoms | 17.75 | 10.37 | 14.27 | 8.61 | 4.38 | 479 | <.001 | 0.36 |
| W3 CB victimization | 1.20 | 1.98 | 1.13 | 2.14 | 0.44 | 585 | .659 | 0.03 |

*Note*. W1 = wave 1; W2 = wave 2; W3 = wave 3; CB = cyberbullying.
